# Early conversion of maternal androgens affects the embryo already in the first week of development

**DOI:** 10.1098/rsbl.2022.0593

**Published:** 2023-03-01

**Authors:** Yuqi Wang, Bernd Riedstra, Ronja Hulst, Roy Noordhuis, Ton Groothuis

**Affiliations:** Groningen Institute for Evolutionary Life Sciences, University of Groningen, Groningen, The Netherlands

**Keywords:** embryo, heart rate, androgens, maternal effects, birds

## Abstract

Maternal androgen exposure has potent effects on offspring development. As substantial levels of maternal androgens are deposited in avian egg yolks, avian eggs are frequently used to study maternal effects, with a strong focus on post-natal development. However, the underlying pathways are largely unknown. Since the hormones are taken up during the embryonic phase, and these are rapidly metabolized by avian embryos into metabolites such as etiocholanolone, we studied the effects of yolk androgens (testosterone and androstenedione) and their metabolite etiocholanolone during the first few days of embryonic development. As embryonic heart rate is often used as an indicator of embryonic development, we measured the heart rate from day 3 to day 6 of incubation by using a shell-less culture technique in rock pigeon eggs (*Columba livia)*. Increased androgen exposure increased heart rate, and increased etiocholanolone mimicked this effect, albeit in a small sample size. This indicates that exposure to maternal androgens increases embryonic overall metabolism which may account for the developmental outcomes found in previous studies such as increased growth. Moreover, etiocholanolone is likely to be an important metabolite in a non-genomic pathway underlying the androgen-mediated maternal effect.

## 1. Introduction

Androgen-mediated maternal effects have been substantially studied across taxa (mammals: [1–3]; fish: [4]; reptiles: [5]). Increased maternal androgens increases offspring growth and competitiveness and levels of maternal androgens vary with the mother's environment [6]. Mothers are therefore believed to differentially expose their embryos to androgens in relation to a range of environmental and maternal factors to adjust offspring phenotypes to their environments. However, the underlying pathways are as yet unclear. Despite the fact that the maternal hormones are taken up by the offspring during the embryonic phase, most studies focus on postnatal effects.

In oviparous species, maternal (yolk) androgens (principally testosterone and androstenedione) are often heavily metabolized by the embryos during very early development into metabolites such as their conjugates and etiocholanolone [5,7]. This raises the question how maternal androgens exert their potent effects while being so rapidly metabolized? So far, this was partly explained by assuming that the hydrophilic conjugates facilitate the uptake of the lipophilic free-form androgens from the yolk into the embryonic circulation where the conjugates are converted back to free forms [8]. However, other metabolites, e.g. etiocholanolone, cannot be converted back [7] and are therefore considered either as diversifying forms that mediate the observed variety of androgen effects (diversification hypothesis) or as a way to inactivate maternal androgens and thus their effects (clearance hypothesis). Supporting the diversification hypothesis; etiocholanolone has a biological function in promoting erythropoiesis [9,10] and neuronal modulation [11], yet does not increase early embryonic growth [12]. Hence, further knowledge of the effects of etiocholanolone on embryonic development is crucial in supporting this hypothesis. Alternatively, elevated etiocholanolone exposure may not affect embryonic development, supporting the clearance hypothesis, which opens the possibility for mother–offspring conflict over exposure to androgens.

Many avian females lay more than one egg per breeding attempt and start incubation before clutch completion [13]. Consequently, later laid eggs hatch later, providing a disadvantage for these chicks in sibling competition [14]. In several species, mothers expose later laid eggs to increased levels of androgens that boost the competitive capacity of hatchlings [15–19]. A recent study in rock pigeon (*Columba livia*) embryos showed that increased maternal androgen levels increased embryonic heart rate during mid-term incubation and a positive relationship between embryonic heart rate and tissue growth at mid-term incubation [20], indicative of an increased developmental rate.

Unfortunately, that study lacked heart rate data before day 6 of incubation, while the metabolites of maternal androgens were already present in embryonic tissues at day 2 and the levels of maternal androgens were substantially decreased [20]. Given that the start of the embryonic heartbeat and blood circulating is usually between 30 to 40 h after the onset of incubation [21,22], it is likely that maternal androgens and/or its metabolites can affect the initial development of the embryonic heart earlier than day 6. As androgen receptors start to become present around the time that androgens are already decreasing, and etiocholanolone has a low affinity to these receptors, this opens the possibility that the androgen affects are mediated via a non-genomic pathway.

To investigate the potential effects of maternal androgens and etiocholanolone on early embryonic heart rate before day 6, we increased yolk testosterone and androstenedione levels, or etiocholanolone levels in rock pigeon eggs [7]. To determine embryonic heart rate, we used a shell-less culture, enabling visual observation of heart rate. We expected that increasing testosterone and androstenedione resulted in increased embryonic heart rate and that increasing etiocholanolone mimicked this effect. Rock pigeons lay two eggs per breeding attempt whereas second-laid eggs contain substantially more maternal androgens than first-laid eggs. Therefore, we also expected that second-laid eggs have higher embryonic heart rates than the first-laid [20].

## 2. Methods

### (a) Housing

Forty pairs of rock pigeons were housed in an outdoor aviary (dimensions: 45 m length × 9.6 m width × 3.75 m high) at the University of Groningen (Groningen, the Netherlands) under natural light and temperature conditions, and ad libitum access to food, grit, and water. Food consisted of a mixture of commercial pigeon seeds (Kasper 6721 and 6712), P40 vitamin supplement (Kasper P40). Fifty nest-boxes (dimensions: 60 cm × 50 cm × 36 cm) were placed on one long side of the aviary in 1-m intervals at a height of 1.5 m and 2.5 m. Boxes were equipped with breeding bowls and nesting materials were provided in the aviary. This experiment was approved by the animal welfare committee of the University of Groningen.

### (b) Egg collection

Eggs were collected in the spring/summer of 2021 and 2022. Boxes were checked twice daily at 12.00 h and 19.00 h to collect freshly laid eggs, which were then individually coded and transported to our inside facility and stored at room temperature for no more than 3 days, after which they were used for hormone manipulation and heart rate monitoring (see below).

### (c) Hormone manipulations

At oviposition, rock pigeon eggs contain high levels of testosterone and androstenedione but barely any etiocholanolone. Whereas the former decrease substantially, the latter substantially increases from oviposition to day 4 of incubation [7]. Therefore, we manipulated yolk androgens at oviposition and yolk etiocholanolone at day 4 of incubation. We used two control groups: control eggs were injected with 50 µl sesame oil either at the onset of (C0) or on day 4 of incubation (C4). Androgen-treated eggs (A4T) were injected with 50 µl sesame oil containing 144 ng ml^−1^ testosterone (T; art. no. 46923-250MG-R, Sigma) and 2210 ng ml^−1^ androstenedione (A4; art. no. 46033-250MG46033-250 mg, Sigma) similar to Wang *et al*., [20]. Eggs assigned to receive etiocholanolone (ETIO) were injected with 50 µl sesame oil containing 1293 ng ml^−1^ etiocholanolone (art. no. NMID551C-1MG, Sigma). Applying these concentrations are within the physiological range (average plus two times the standard deviation, based on Kumar *et al*., [7]). In total, there were 76 C0, 81 A4T, 45 to the C4 and 24 ETIO eggs (due to scarcity of etiocholanolone solutions (see electronic supplementary material, table S1A for the sample size per year and egg laying sequence)).

### (d) Embryo culture and egg incubation

In year 2021, we used the embryo shell-less culture developed by Hamamichi & Nishigori [23]. Briefly, eggs were disinfected using a 70% ethanol spray and air dried in an air-flow cabinet. Eggs were then placed horizontally for 5 min, allowing the embryo/embryonic disc to float to the upper side of the egg to reduce the risk of damage when eggs were subsequently transferred into a transparent plastic cup by cracking the underside against the edge of the cup. Subsequently, all eggs were randomly assigned to one of the four treatments. We then injected the egg yolks of the C0 and A4T group with the appropriate solutions using a disposable insulin syringe (U-100, 29G needle × 12.7 mm, BD Micro-Fine). The cups were then sealed using clear polyethylene film and a rubber band and placed in an incubator (Maxi II Eco, Brinsea, US) set at 37.5°C and 55% relative humidity. The cups containing C4 and ETIO eggs were also sealed by clear polyethylene film and rubber bands and put into the incubator until day 4 of incubation when they were injected with their corresponding solution. The transparent cups enable a continuous observation of the heartbeat and heart structural development, yet this method also causes a high mortality rate of the embryos (see electronic supplementary material, table S1). Therefore, an alternative method was used in year 2022. Briefly, freshly collected eggs were placed horizontally for 5 min, allowing yolk to float to the upper side. The eggshell was disinfected with 70% ethanol and a small whole was drilled in the eggshell on the top side, approximately 2 mm beside the horizontal central axis, and approximately at two-third of the egg toward the air chamber using a drill (Dremel TM). We then injected the egg yolks of the C0 and A4T group with the syringe inserting through the hole with an angle of approximately 15°. A drop of Vetbond (3 M, USA) was applied to seal the hole after injection. Directly after the treatment, eggs were placed in an incubator with the same setting as year 2021. Eggs of C4 and ETIO group were incubated intact until day 4 of incubation when the treatment was conducted to these two groups.

### (e) Embryonic heart rate measurement

Embryos were checked daily and undeveloped eggs were discarded. Embryonic heartbeat was detectable by eye from day 3 after the onset of incubation onwards. When heart rate was measured the eggs were temperately removed from the incubator and video recorded for 1 min with a smart phone. The shell-less cultured embryos in 2021 were kept in the incubator as long as day 7 when all embryos were terminated by placing them in a −20°C freezer. In 2022, a small window was created in the blunt side of the egg for observation at the day of heart rate measurement. When the position of the embryo blocked the view of the heart, the egg was carefully cracked into a Petri dish and only the first 20 s of the video recordings were used for measurement as the embryo temperature decreased quickly causing a decreasing heart rate. Video recordings were then analysed by playing the video 0.5 times speed ad counting the number of beats per time interval. In total, we obtained heart rates of 13 C0, 18 A4T, 12 C4 and five ETIO embryos (see electronic supplementary material, table S1B for the sample size per year and egg laying sequence). The survival of control and hormone-treated eggs did not differ (ANOVA, *F* = 0.13, *p* = 0.94) and survival did not differ between years or egg laying sequence (ANOVA, both *F* < 0.42, *p* > 0.52) which was tested by linear regression with egg laying sequence and year as covariates.

### (f) Statistical analyses

To test whether hormone treatment affected embryonic heart rate, linear mixed models (LMMs) were used with heart rate as dependent variable and treatment, embryo age and the interaction of treatment and embryo age as independent variables. Furthermore, egg laying sequence and year were added as covariates and egg identity as random effect. Since the heart rate from C0 embryos did not differ from C4 embryos (LMM, *F* = 0.21, *p* = 0.65), we merged these groups (CTL group) to increase statistical power. To address our specific predictions, three LMMs were built to compare embryonic heart rate between (1) CTL and A4T embryos; (2) CTL and ETIO embryos; and (3) A4T and ETIO embryos. Due to the low sample size (see electronic supplementary material, table S1) we excluded ‘year’ from the latter two models to increase statistical power. Statistical analyses were performed in R, v. 2.10.1 [24].

## 3. Results

### (a) Effect on embryonic heart rate

**Figure 1.**
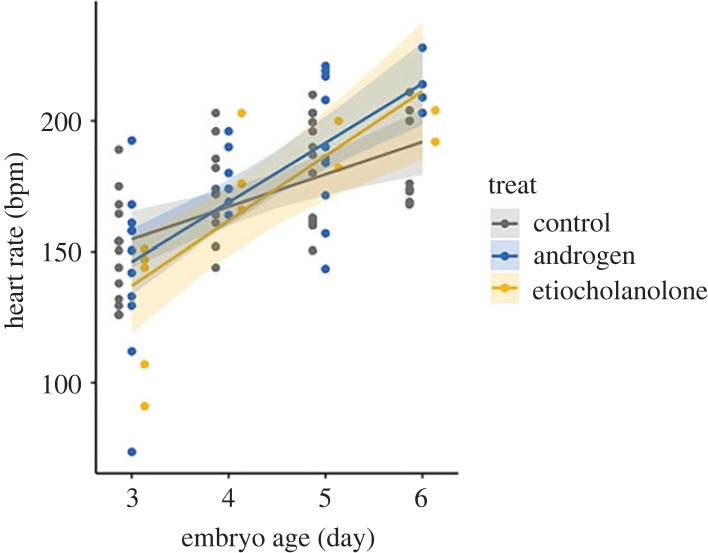
Embryonic heart rate (beats per minute, bpm) of eggs with different treatments. Each dot represents an observed raw data value, with the estimated trend lines and 95% CI bands generated from a linear mixed model (LMM), with treatment, embryo age, the interaction of treatment and embryo age as independent variables, egg laying sequence covariate, and egg ID as random effect.

Embryonic heart rate increased with embryo age in all groups (figure 1, LMMs, all *F* > 41.2, *p* < 0.001). The A4T group had an increased heart rate compared to the CTL group (LMM, *F* = 4.23, *p* = 0.04) and the interaction effect between treatment and embryonic age was significant (LMM, *F* = 4.10, *p* = 0.05). Albeit a small sample size, the ETIO group also showed an increased heart rate compared to the CTL group (LMM, *F* = 4.46, *p* = 0.04) and was also dependent on embryo age (LMM, *F* = 4.00, *p* = 0.05). There was no difference between the A4T and ETIO groups (LMM, *F* = 0.04, *p* = 0.84). Egg laying sequence or year did not show an effect in any of the models (LMMs, all *F* < 0.79, all *p* > 0.40).

## 4. Discussion

In this study, we focused on the effects of maternal testosterone, androstenedione and etiocholanolone, the latter being an important metabolite of testosterone and androstenedione, on early embryonic heart rate. Embryos exposed to increased yolk testosterone and androstenedione, and to etiocholanolone, showed a steeper increase in heart rate over the first 6 days of development than the control group. To our knowledge, this is the first study that tested the effect of maternal androgens during this early prenatal phase and shows that a metabolite of maternal androgens (etiocholanolone) mimics an effect induced by the maternal androgens.

We acknowledge that our sample size is small but we, as well as a statistician acting as referee, are of the opinion that it is sufficiently reliable for interpreting our results, as our analysis has several features (low sample sizes, aggregation of covariate groups, random effects) that could have yielded non-significant results (i.e. obscured any signal in the data) which has not happened. Also, although the number of embryos is somewhat low, the interest is in the replicate observations of each embryo, for which we also took their dependency into account by embryo ID as random effect.

It is unlikely that this effect is achieved via hormone receptors as androgen receptors are only starting to become expressed by embryos around day 4–5 of incubation [25,26]. At this stage, maternal androgens have already been heavily metabolized and one of the main metabolites, etiocholanolone, only has a low affinity to androgen receptors [27]. This indicates that the observed effect may be mediated by a rapid androgen-induced non-genomic pathway that does not involve regulation of gene transcription [28–30]. The presence of maternal androgens, etiocholanolone, and other yet unknown androgens in embryonic tissues as early as day 2 of incubation in this same species [20] gives indirect evidence that such a non-genomic pathway may play a key role in early embryonic development. Therefore, it is promising to further study the effects of maternal androgen metabolites during early embryonic development.

Lastly, irrespectively of treatment, we did not find an effect of egg laying sequence on embryonic heart rate. This was unexpected since we previously showed that embryos from second-laid eggs had higher heart rates than embryos from first-laid eggs from day 6 to day 10 of incubation [20]. Assuming that that effect was caused by differential investment in growth in different developmental stages (depending on egg sequence), it is possible that before day 6 other energy-costing processes such as the development of the neural circuitry may constrain increasing heart rate.

In conclusion, our results indicate that maternal androgens have a stimulatory effect on embryonic heart rate already during the first 6 days of incubation and that this effect is likely to be mediated by etiocholanolone via a non-genomic pathway. The underlying adaptive significance may be that through embryonic metabolism, maternal androgens diversify into multiple metabolites that could eventually exert pleiotropic effects. We encourage future studies exploring not only the biological functions of other metabolites of maternal androgens but to also relate these to post-natal development and their evolutionary consequences so that we build a more complete view of the androgen-mediated maternal effects from both a proximate and ultimate perspective.

## Data Availability

The datasets supporting this article have been uploaded as part of the electronic supplementary material [31].

## References

[1] Edwards PD, Lavergne SG, McCaw LK, Wijenayake S, Boonstra R, McGowan PO, Holmes MM. 2021 Maternal effects in mammals: broadening our understanding of offspring programming. Front Neuroendocrinol. **62**, 100924. (10.1016/j.yfrne.2021.100924) 33992652

[2] Gil D. 2008 Chapter 7 Hormones in avian eggs: physiology, ecology and behavior. In Advances in the study of behavior, pp. 337-398. London, UK: Academic Press.

[3] Groothuis TGG, Hsu B-Y, Kumar N, Tschirren B. 2019 Revisiting mechanisms and functions of prenatal hormone-mediated maternal effects using avian species as a model. Phil. Trans. R. Soc. B **374**, 20180115. (10.1098/rstb.2018.0115) 30966885 PMC6460091

[4] Schaafsma SM, Groothuis TGG. 2012 Sex-specific effects of maternal testosterone on lateralization in a cichlid fish. Anim. Behav. **83**, 437-443. (10.1016/j.anbehav.2011.11.015)

[5] Paitz RT, Bowden RM. 2009 Rapid decline in the concentrations of three yolk steroids during development: is it embryonic regulation? Gen. Comp. Endocrinol. **161**, 246-251. (10.1016/j.ygcen.2009.01.018) 19523390

[6] Groothuis TGG, Müller W, von Engelhardt N, Carere C, Eising C. 2005 Maternal hormones as a tool to adjust offspring phenotype in avian species. Neurosci. Biobehav. Rev. **29**, 329-352. (10.1016/j.neubiorev.2004.12.002) 15811503

[7] Kumar N, van Faassen M, Kema I, Gahr M, Groothuis TGG. 2018 Early embryonic modification of maternal hormones differs systematically among embryos of different laying order: a study in birds. Gen. Comp. Endocrinol. **269**, 53-59. (10.1016/j.ygcen.2018.08.014) 30110617

[8] Paitz RT, Bowden RM. 2013 Sulfonation of maternal steroids is a conserved metabolic pathway in vertebrates. Integr. Comp. Biol. **53**, 895-901. (10.1093/icb/ict027) 23620254

[9] Levere RD, Kappas A, Granick S. 1967 Stimulation of hemoglobin synthesis in chick blastoderms by certain 5beta androstane and 5beta pregnane steroids. Proc. Natl Acad. Sci. USA **58**, 985-990. (10.1073/pnas.58.3.985) 5233854 PMC335736

[10] Irving RA, Mainwaring WIP, Spooner PM. 1976 The regulation of haemoglobin synthesis in cultured chick blastoderms by steroids related to 5β-androstane. Biochem. J. **154**, 81-93. (10.1042/bj1540081) 1275915 PMC1172679

[11] Mouton JC, Duckworth RA. 2021 Maternally derived hormones, neurosteroids and the development of behaviour. Proc. R. Soc. B **288**, 20202467. (10.1098/rspb.2020.2467) PMC7893274 33499795

[12] Campbell NA, Angles R, Bowden RM, Casto JM, Paitz RT. 2020 Characterizing the timing of yolk testosterone metabolism and the effects of etiocholanolone on development in avian eggs. J. Exp. Biol. **223**, jeb210427. (10.1242/jeb.210427) 32001543

[13] Lack D. 1968 Ecological adaptations for breeding in birds. London, UK: Methuen and Co.

[14] Magrath RD. 1990 Hatching asynchrony in altricial birds. Biol. Rev. **65**, 587-622. (10.1111/j.1469-185X.1990.tb01239.x)

[15] Schwabl H. 1993 Yolk is a source of maternal testosterone for developing birds. Proc. Natl Acad. Sci. USA **90**, 11 446-11 450. (10.1073/pnas.90.24.11446) 8265571 PMC48000

[16] Eising CM, Groothuis TGG. 2003 Yolk androgens and begging behaviour in black-headed gull chicks: an experimental field study. Anim. Behav. **66**, 1027-1034. (10.1006/anbe.2003.2287)

[17] Eising CM, Eikenaar C, Schwabl H, Groothuis TGG. 2001 Maternal androgens in black-headed gull (*Larus ridibundus*) eggs: consequences for chick development. Proc. R. Soc. B **268**, 839-846. (10.1098/rspb.2001.1594) PMC1088678 11345330

[18] Müller MS, Moe B, Groothuis TGG. 2013 Testosterone increases siblicidal aggression in black-legged kittiwake chicks (*Rissa tridactyla*). Behav. Ecol. Sociobiol. **68**, 223-232. (10.1007/s00265-013-1637-z)

[19] Merkling T et al. 2016 Maternal effects as drivers of sibling competition in a parent–offspring conflict context? An experimental test. Ecol. Evol. **6**, 3699-3710. (10.1002/ece3.1777) 28725354 PMC5513303

[20] Wang Y, Riedstra B, Groothuis T. 2023 Embryonic heart rate is affected by yolk androgens and egg laying sequence, and correlates with embryonic tissue growth: a study in rock pigeons. Gen. Comp. Endocrinol. **333**, 114213. (10.1016/j.ygcen.2023.114213) 36642229

[21] Bogue JY. 1932 The heart rate of the developing chick. J. Exp. Biol. **9**, 351-358. (10.1242/jeb.9.4.351)

[22] Hamburger V, Hamilton HL. 1951 A series of normal stages in the development of the chick embryo. Dev. Dyn. **195**, 231-272. (10.1002/aja.1001950404) 1304821

[23] Hamamichi S, Nishigori H. 2001 Establishment of a chick embryo shell-less culture system and its use to observe change in behavior caused by nicotine and substances from cigarette smoke. Toxicol. Lett. **119**, 95-102. (10.1016/S0378-4274(00)00300-3) 11311570

[24] R Core Team. 2009 R: a language and environment for statistical computing. Vienna, Austria: R Foundation for Statistical Computing. See http://www.R-project.org/.

[25] Endo D, Murakami S, Akazome Y, Park MK. 2007 Sex difference in Ad4BP/SF-1 mRNA expression in the chick-embryo brain before gonadal sexual differentiation. Zoolog. Sci. **24**, 877-882. (10.2108/zsj.24.877) 17960991

[26] Kumar N, Lohrentz A, Gahr M, Groothuis TGG. 2019 Steroid receptors and their regulation in avian extraembryonic membranes provide a novel substrate for hormone mediated maternal effects. Sci. Rep. **9**, 1-6. (10.1038/s41598-019-48001-x) 31395925 PMC6687743

[27] Gao W, Bohl CE, Dalton JT. 2005 Chemistry and structural biology of androgen receptor Wenqing. Chem. Rev. **68**, 1-9. 10.1021/cr020456u PMC2096617 16159155

[28] Foradori CD, Weiser MJ, Handa RJ. 2008 Non-genomic actions of androgens. Front Neuroendocrinol. **29**, 169-181. (10.1016/j.yfrne.2007.10.005) 18093638 PMC2386261

[29] Belelli D, Lambert JJ. 2005 Neurosteroids: endogenous regulators of the GABAA receptor. Nat. Rev. Neurosci. **6**, 565-575. (10.1038/nrn1703) 15959466

[30] Schverer M, Lanfumey L, Baulieu EE, Froger N, Villey I. 2018 Neurosteroids: non-genomic pathways in neuroplasticity and involvement in neurological diseases. Pharmacol. Ther. **191**, 190-206. (10.1016/j.pharmthera.2018.06.011) 29953900

[31] Wang Y, Riedstra B, Hulst R, Noordhuis R, Groothuis T. 2023 Early conversion of maternal androgens affects the embryo already in the first week of development. Figshare. (10.6084/m9.figshare.c.6442424) PMC9975654 36855858

